# The R.B.N.A. and the College

**Published:** 1917-11-17

**Authors:** M. S. Rundle

**Affiliations:** Secretary College of Nursing, Ltd. 6 Vere Street, W. 1


### THE EDITOR'S LETTER-BOX.

### The R.B.N.A. and the College.

THE VIEWS OF THE COUNCIL OF THE COLLEGE.

We have received the following letter for publication :—

To The Secretary, The Royal British Nurses' Association, 10 Orchard Street, W.

Dear Madam.—In view of the delay and inconvenience resulting from the course of action pursued by your Council, the Council of the College has carefully considered whether it is now in the interests of its members to proceed further with the scheme for the amalgamation of the two Associations.

After nearly a year's experience of the dilatory manner in which proceedings have been conducted by your Association with the Privy Council, my Council has reluctantly reached the conclusion that there is at the present time little evidence of any real desire on the part of your Council to amalgamate with the College, even though the project of amalgamation was welcomed by your members, and we have conceded everything asked by you. The alterations suggested by the Privy Council in the Supplemental Charter and Bye-Laws do not in our judgment modify that document as agreed between us in any substantial sense.

The Charter of the Royal British Nurses' Association, as it stands, gives to your Corporation powers which are admittedly quite inadequate to the present needs of the nursing profession, and the Supplemental Charter was, therefore, a necessity, if the Royal British College of Nursing was to possess such powers as the College already has under its Memorandum and Articles of Association.

Anxious as my Council is that the College of Nursing should be established at the earliest possible date on a representative basis, it nevertheless, in its desire to meet the demands of your Association, consented to accept the proviso that, as a condition of the amalgamation, the joint Council set out in the Supplemental Charter should hold office for two years. This has already proved a serious impediment to the College in its policy of securing State Registration for Nurses, and my Council prefers to abide by the terms of its own constitution, under which the Council becomes elective in April next.

It was undoubtedly of some importance to the College in its early days to seek to ally itself with your Association, just as it was of importance for your Association to obtain enlarged powers, the new infusion of energy, and increased membership, which would have come about by the establishment of the amalgamated bodies as the Royal British College of Nursing; but as the College of Nursing has now become well known, and as an appeal for funds made by the British Women's Hospital on its behalf has met with the widest sympathetic reception by the press and public, the advantage to the College of amalgamation becomes less obvious. If, however, the terms of our agreement with your Association are fulfilled, the College is prepared to take the further steps necessary to give effect to the amalgamation.

Should this not come about, then I am instructed to ask your Council to refrain from incurring further expenditure, which, as you will no doubt remember, the College has to find, and although the agreement between us cannot be legally terminated until next December, to regard negotiations as practically at an end.

In conclusion, my Council instructs me to say how sincerely it regrets that its hopes of union with your Association should have been frustrated, and that it should not have the help it anticipated from the members of your Association whom it hoped to welcome on the Council of the Royal British College of Nursing in prosecuting the important tasks which have already been delayed, in the hopes of a joint agreement and professional unity.—I am, dear Madam, yours faithfully,

(Signed) M. S. Rundle,

Secretary College of Nursing, Ltd.

6 Vere Street, W. 1, November 3, 1917.

**For comment, see p. 148.**

